# Glutamate as a neurotransmitter in the healthy brain

**DOI:** 10.1007/s00702-014-1180-8

**Published:** 2014-03-01

**Authors:** Y. Zhou, N. C. Danbolt

**Affiliations:** The Neurotransporter Group, Department of Anatomy, Institute of Basic Medical Sciences, University of Oslo, Blindern, P.O. Box 1105, 0317 Oslo, Norway

**Keywords:** Glutamate uptake, EAAT2, EAAT1, EAAT3, slc1a2, slc1a1, slc1a3, Glutamate, Glutamate transporter, Immunocytochemistry, Neuropil

## Abstract

Glutamate is the most abundant free amino acid in the brain and is at the crossroad between multiple metabolic pathways. Considering this, it was a surprise to discover that glutamate has excitatory effects on nerve cells, and that it can excite cells to their death in a process now referred to as “excitotoxicity”. This effect is due to glutamate receptors present on the surface of brain cells. Powerful uptake systems (glutamate transporters) prevent excessive activation of these receptors by continuously removing glutamate from the extracellular fluid in the brain. Further, the blood–brain barrier shields the brain from glutamate in the blood. The highest concentrations of glutamate are found in synaptic vesicles in nerve terminals from where it can be released by exocytosis. In fact, glutamate is the major excitatory neurotransmitter in the mammalian central nervous system. It took, however, a long time to realize that. The present review provides a brief historical description, gives a short overview of glutamate as a transmitter in the healthy brain, and comments on the so-called glutamate–glutamine cycle. The glutamate transporters responsible for the glutamate removal are described in some detail.

## Introduction

Outside the community of biomedical scientists, glutamate is probably best known as “monosodium glutamate” or “MSG” which is the sodium salt of glutamic acid and a white crystalline solid used as a flavor or taste enhancer in food (food additive number E620). This, however, is not the reason for the enormous scientific interest in glutamate. The main motivation for the ongoing worldwide research on glutamate is that glutamate is the major excitatory transmitter in the brain.

Like other signaling substances, the signaling effect of glutamate is not dependent on the chemical nature of glutamate, but on how cells are programmed to respond when exposed to it. Because the glutamate receptor proteins are expressed on the surface of the cells in such a way that they can only be activated from the outside, it follows that glutamate exerts its neurotransmitter function from the extracellular fluid. Consequently, control of receptor activation is achieved by releasing glutamate to the extracellular fluid and then removing glutamate from it. Because there are no enzymes extracellularly that can degrade glutamate, low extracellular concentrations require cellular uptake. This uptake is catalyzed by a family of transporter proteins located at the cell surface of both astrocytes and neurons (e.g. Danbolt 2001; Grewer and Rauen 2005; Tzingounis and Wadiche 2007; Vandenberg and Ryan 2013).

Because glutamate is the major mediator of excitatory signals as well as of nervous system plasticity, including cell elimination, it follows that glutamate should be present at the right concentrations in the right places at the right time. It further follows that cells should have the correct sensitivity to glutamate and have energy enough to withstand normal stimulation, and that glutamate should be removed with the appropriate rates from the right locations. Both too much glutamate and too little glutamate are harmful. Excessive activation of glutamate receptors may excite nerve cells to their death in a process now referred to as “excitotoxicity”. This toxicity was initially perceived as a paradox like “Dr. Jekyll and Mr. Hyde”, but it is now clear that glutamate is toxic, not in spite of its importance, but because of it. As outlined before (Danbolt 2001), the intensity of glutamatergic stimulation that a given cell can tolerate, depends on several factors. As long as one variable is not extreme, it will be the combination of several factors that will determine the outcome.

It took a long time to realize that glutamate is a neurotransmitter in part because of its abundance in brain tissue and in part because it is at the crossroad of multiple metabolic pathways (e.g. Erecinska and Silver 1990; Broman et al. 2000; McKenna 2007; Hertz 2013). There is 5–15 mmol glutamate per kg brain tissue, depending on the region, more than that of any other amino acid (Schousboe 1981). So although it was noted early on that glutamate plays a central metabolic role in the brain (Krebs 1935), that brain cells have a very high glutamate uptake activity (Stern et al. 1949) and that glutamate has an excitatory effect (Hayashi 1954; Curtis et al. 1959, 1960), the transmitter role was not realized until the early 1980s (for review see Fonnum 1984).

In fact, glutamate metabolism is complex and compartmentalized (Berl et al. 1961, 1962; Van den Berg and Garfinkel 1971; Balcar and Johnston 1975). The important role of glutamate uptake in the control of the excitatory action of glutamate was recognized (Logan and Snyder 1971, 1972; Wofsey et al. 1971; Balcar and Johnston 1972). This became a hot research topic. A number of different glutamate and aspartate analogues were synthesized, and heterogeneity within glutamate uptake was uncovered suggesting more than one uptake mechanism (Ferkany and Coyle 1986; Robinson et al. 1991, 1993; Fletcher and Johnston 1991; Balcar and Li 1992; Rauen et al. 1992).

Similarly, several families of glutamate receptor proteins were identified with molecular cloning (for review see Niswender and Conn 2010; Traynelis et al. 2010; Nicoletti et al. 2011). The receptors were classified as *N*-methyl-d-aspartate (NMDA) receptors (Gonda 2012; Bonaccorso et al. 2011; Santangelo et al. 2012), AMPA (α-amino-3-hydroxy-5-methyl-4-isoxazole propionic acid) receptors (Rogawski 2013), kainate receptors (Lerma and Marques 2013) and metabotropic receptors (Gregory et al. 2013). Most, if not all, cells in the nervous system express at least one type of glutamate receptor (Steinhauser and Gallo 1996; Vernadakis 1996; Forsythe and Barnes-Davies 1997; Wenthold and Roche 1998; Petralia et al. 1999; Conti et al. 1999; Shelton and McCarthy 1999; Bergles et al. 2000). The locations and functional properties of each type are beyond the scope of this review.

Medicinal chemists continued to synthesize new compounds and it is now possible to differentiate pretty well between the various receptors and transporters. Considering the relatively large number of proteins with ability to bind glutamate, it may seem strange that it is possible to find compounds that can distinguish between them. The reason is the high flexibility of the glutamate molecule which permits several conformations that are only minimally less favorable energetically at body temperature than the lowest energy conformation (Bridges et al. 1991). This implies that glutamate can take many shapes and explains, in part, why the various glutamate binding proteins (transporters, receptors, enzymes) can have quite different binding sites and still be able to bind glutamate. A large number of compounds are now available, and there are a number of excellent reviews on the topic (e.g. Bräuner-Osborne et al. 1997; Jensen and Bräuner-Osborne 2004; Shigeri et al. 2004; Ritzen et al. 2005; Thompson et al. 2005; Bridges and Esslinger 2005; Shimamoto 2008; Bridges et al. 2012a, b; Gregory et al. 2013; Gonda 2012; Bonaccorso et al. 2011).

## Identification of plasma membrane glutamate transporters

A glutamate transporter, now known as EAAT2 (GLT-1; slc1a2; Pines et al. 1992), was purified in an active form from rat brain by employing reconstitution of transport as the assay to monitor the purification process (Danbolt et al. 1990). The purification was based on solubilization of rat brain membranes with a detergent and fractionation by conventional chromatographic techniques. This resulted in a 30-fold increase in specific activity, but due to inactivation, the purification ratio was closer to 100-fold. It was hard to convince ourselves that this moderate enrichment was sufficient to yield a pure preparation, and it was even harder to convince others. The fact that the protein tends to give wide bands in electrophoresis gels did not make the task any easier (see Danbolt 1994). Nevertheless, this was a pure preparation (Levy et al. 1993; Lehre and Danbolt 1998). Antibodies were raised to the purified protein and used to localize it in the brain (Danbolt et al. 1992; Levy et al. 1993) and to screen expression libraries. The sequence of the isolated cDNA predicted correctly a protein of 573 amino acids (Pines et al. 1992). Simultaneously, but independently of each other, three other research teams succeeded in cloning another two glutamate transporters using completely different approaches. Storck et al. (1992) were purifying a galactosyltransferase from rat brain and observed that a 66 kDa hydrophobic glycoprotein copurified with this protein. The purified protein was subjected to limited proteolysis. Partial amino acid sequences were obtained and used for synthesizing degenerate oligonucleotide probes for screening of a rat brain cDNA library. This resulted in the identification of 543 amino acid residues long protein now referred to as EAAT1 (GLAST; slc1a3; Storck et al. 1992). EAAT3 (EAAC1; slc1a1) was isolated from a rabbit jejunum by *Xenopus laevis* oocyte expression cloning (Kanai and Hediger 1992). The cDNA sequence contains an open reading frame coding for a protein of 524 amino acids. The rat brain equivalent is 89.9 % identical and 523 amino acids long (Kanai et al. 1993; Bjørås et al. 1996). The three human counterparts were quickly identified and named excitatory amino acid transporter (EAAT)1–3 (Arriza et al. 1994). Another two glutamate transporters were found later: EAAT4 (Fairman et al. 1995) and EAAT5 (Arriza et al. 1997). All the EAATs catalyze coupled transport of 1H^+^, 3Na^+^, and 1K^+^ with one substrate molecule (Klöckner et al. 1993; Zerangue and Kavanaugh 1996a; Levy et al. 1998; Owe et al. 2006). l-Glutamate and dl-aspartate are transported with similar affinities while d-glutamate is not. It is important to note that the transporters are performing exchange in addition to net uptake. Exchange is a process whereby the transporters exchange external and internal substrate molecules in a 1:1 relationship (see Fig. 5 in Danbolt 2001). Thus, when transportable uptake inhibitors are added to cell cultures, the inhibitors induce glutamate release from the cells (e.g. Volterra et al. 1996; Danbolt 2001) Table 1.

**Table 1:** Overview of the nomenclature of plasma membrane glutamate transporters

HUGO name	Other names
Excitatory amino acid transporter 1 (EAAT1; slc1a3)	GLAST (Storck et al. 1992; Tanaka 1993b; Arriza et al. 1994)
Excitatory amino acid transporter 2 (EAAT2; slc1a2)	GLT-1; GLT1 (Pines et al. 1992; Arriza et al. 1994)
Excitatory amino acid transporter 3 (EAAT3; slc1a1)	EAAC1 (Kanai and Hediger 1992; Arriza et al. 1994)
Excitatory amino acid transporter 4 (EAAT4; slc1a6)	(Fairman et al. 1995)
Excitatory amino acid transporter 5 (EAAT5; slc1a7)	(Arriza et al. 1997)

Glutamate transporters belong to the solute carrier (slc) family 1 (high-affinity glutamate and neutral amino acid transporter family; Hediger et al. 2013). Although there are several proteins with ability to transport glutamate, the term “glutamate transporter” is usually used to describe the five “high-affinity glutamate transporters” also called “excitatory amino acid transporters (EAATs)”. The actual meanings of the acronyms (*GLAST* glutamate–aspartate transporter, *GLT1* glutamate transporter, *EAAC* excitatory amino acid carrier, *EAAT* excitatory amino acid transporter) are not important, as they do not reflect functional differences among the transporters. The nomenclature used here is the one adopted by the HUGO Gene Nomenclature Committee (Hediger et al. 2013)

The substrate selectivities are not reviewed here. We will only point out (a) that the commonly used uptake inhibitor dihydrokainate (DHK; CAS 52497-36-6) blocks EAAT2 with high selectivity over the other EAATs (Arriza et al. 1994; Bridges et al. 1999), and (b) that dl-*threo-β*-benzyloxyaspartate (TBOA; CAS 205309-81-5) and its variants (e.g. PMB-TBOA and TFB-TBOA) block all the five EAATs (Bridges et al. 1999; Shimamoto 2008). These compounds are competitive inhibitors that are not transportable. This implies that they block both uptake and exchange (for a detailed explanation, see sect. 6.5 in Danbolt 2001). For more information, we recommend the outstanding review by Bridges et al. (1999) as an introduction and more recent reviews for the last updates (e.g. Jensen and Bräuner-Osborne 2004; Shigeri et al. 2004; Bridges and Esslinger 2005; Shimamoto and Shigeri 2006; Shimamoto 2008; Sagot et al. 2008).

The EAAT-type of transporters also functions as chloride channels (Fairman et al. 1995; Zerangue and Kavanaugh 1996a; Wadiche et al. 1995a, b; Ryan and Mindell 2007; Takayasu et al. 2009). EAAT4 and EAAT5 have the largest chloride conductance (Mim et al. 2005; Gameiro et al. 2011), and may function more as inhibitory glutamate receptors than as transporters (Dehnes et al. 1998; Veruki et al. 2006; Schneider et al. 2014). Arachidonic acid elicits a substrate-gated proton current associated with the glutamate transporter EAAT4 (Fairman et al. 1998; Tzingounis et al. 1998). In addition, a general feature of sodium coupled transport appears to be transport of water (MacAulay et al. 2001, 2004).

Even though the mammalian transporters have not yet been crystallized, we already know quite a lot about their complex structure (Kanner 2007; Gouaux 2009; Kanner 2013; Vandenberg and Ryan 2013). The EAAT2 and EAAT3 proteins are believed to be homotrimers where the subunits are non-covalently connected (Haugeto et al. 1996). This is in agreement with studies of glutamate transporters from *Bacillus Caldotenax* and *Bacillus stearothermophilus* (Yernool et al. 2003) although crosslinking studies of the mammalian transporters indicate that there may be differences between the EAAT subtypes (Dehnes et al. 1998). These proteins are integral membrane proteins and they depend on the lipid environment, and are influenced by fatty acids such as arachidonic acid (Barbour et al. 1989; Trotti et al. 1995; Zerangue et al. 1995) and by oxidation (Trotti et al. 1996; Trotti et al. 1998). The recent determination of the crystall structure of a glutamate transporter homologue (GltPh) from *Pyrococcus horikoshii* (Yernool et al. 2004) and other transporters (Penmatsa and Gouaux 2013) implies a milestone similar to the cloning of the first transporters in the early 1990s and the generation of knockout mice in the late 1990s. GltPh appear to be a bowl-shaped trimer with a solvent-filled extracellular basin extending halfway across the membrane bilayer. At the bottom of the basin are three independent binding sites (Yernool et al. 2004). This structure is, as uncovered recently, ideal to facilitate rapid transport (Leary et al. 2011).

## The glutamate-cystine exchanger

Another transporter that has got quite a lot of attention lately is the so called glutamine-cystine exchanger (xCT; slc7a11). This transporter was first described in human fibroblasts as an electroneutral 1:1 cystine-glutamate exchanger that carries cystine into the cell in exchange for internal glutamate (Bannai 1986). Thus, the physiological role of this transporter is to act as a cystine transporter that uses the transmembrane gradient of glutamate as driving force. It follows from this that extracellular glutamate inhibits uptake of cystine and that uptake of cystine causes glutamate release. The transporter responsible for this uptake has been identified by molecular cloning (Sato et al. 1999). It is a heterooligomer consisting of two different subunits: the 4F2hc surface antigen (slc3a2) the xCT protein (slc7a11). The substrate selectivities are excellently reviewed by Bridges et al. (2012a, b).

There are several reasons why xCT has become a hot topic (Conrad and Sato 2012; Lewerenz et al. 2013; Bridges et al. 2012a, b). The first important observation was that glioma express high levels of xCT and low levels of EAATs suggesting that they release glutamate and that glutamate toxicity may be a mechanism facilitating their invasion of normal tissue (e.g. Ye et al. 1999; Sontheimer 2004; Takeuchi et al. 2013). Another reason for the interest is that cystine is a source of cysteine needed for synthesis of glutathione (Dringen 2000). There are, however, a number of transporters that can transport cysteine. These comprise EAAT3 (Zerangue and Kavanaugh 1996b), the two alanine-serine-cysteine transporters (Arriza et al. 1993; Shafqat et al. 1993; Hofmann et al. 1994), ASCT1 (slc1a4) and ASCT2 (slc1a5) as well as several others (Bröer 2008). So if cystine is reduced to cysteine at the cell surface, then cysteine can be taken up independently of xCT. Nevertheless, xCT-deficient mice display redox imbalance suggesting that xCT does play a role in glutathione production (Sato et al. 2005). A third reason for the interest in xCT is that xCT has been suggested to be a major source of extracellular glutamate (Baker et al. 2002). This has been highly controversial, but a recent paper based on the xCT-deficient mice is supporting the idea (De Bundel et al. 2011). There are, however, a number of unresolved issues. The distribution of xCT in the brain has not yet been definitively determined, and available data suggest low levels (Sato et al. 2002). If the above observations are due to direct actions of xCT, then there must be enough xCT molecules present to perform the proposed functions. Thus, both the expression levels and the speed which xCT operates (translocation cycles per second per xCT molecule) are important to determine. As xCT is highly inducible (Sato et al. 2001, 2004), it is should be kept in mind that expression levels may change in stressful situations. Finally, if xCT exchanges glutamate and cystine in a 1:1 relationship, then a massive glutamate release can only be mediated by xCT if there is a similar transport of cystine (or another substrate) in the other direction. In conclusion, more work is needed before we fully understand the roles that xCT plays.

## Intracellular glutamate carriers

When glutamate enters the cytoplasm, it may undergo further redistribution to mitochondria or synaptic vesicles (Erecinska and Silver 1990; Nicholls 1993). (A) Mitochondrial glutamate transport: Several of the enzymes for which glutamate is a substrate are located in mitochondria. In agreement, mitochondria possess mechanisms for glutamate translocation. In fact, there are four different carriers: AGC1 (Slc25a12; aralar1; del Arco and Satrustegui 1998), AGC2 (Slc25a13; citrin; aralar2; Kobayashi et al. 1999; Yasuda et al. 2000), GC1 (Slc25a22; Fiermonte et al. 2002) and GC2 (Slc25a18; Fiermonte et al. 2002).

These transporters are very different from the glutamate transporters in the plasma membranes and will not be discussed further here (for review see Palmieri 2013). (B) Glutamate transporters in synaptic vesicles: In glutamatergic nerve terminals, glutamate is carried into synaptic vesicles by means of the so called vesicular glutamate transporters (VGLUTs). These are also very different from those in the plasma membrane (for review see El Mestikawy et al. 2011; Omote et al. 2011) by being independent of sodium and potassium, and by having lower affinity (km around 1 mM). There are three different isoforms: VGLUT1 (Slc17a7; Ni et al. 1994; Bellocchio et al. 1998, 2000; Takamori et al. 2000), VGLUT2 (Slc17a6; DNPI; Aihara et al. 2000) and VGLUT3 (Slc17a8; Takamori et al. 2002).

## Release of glutamate

Glutamate is continuously being released to the extracellular fluid, and inhibition of glutamate uptake leads to extracellular buildups of glutamate within seconds (Jabaudon et al. 1999). Although most of the focus has been on synaptic release of glutamate from nerve terminals by exocytosis of synaptic vesicles, this is not the only mechanism able to supply the extracellular fluid with glutamate (Danbolt 2001). In fact, there are several different non-vesicular (non-exocytotic) mechanisms that appear to be important. One is through anion channels (Kimelberg et al. 1990; Kimelberg and Mongin 1998; Wang et al. 2013) and another is via reversed operation of the glutamate transporting proteins at the plasma membrane (e.g. Levi and Raiteri 1993; Longuemare and Swanson 1995; Roettger and Lipton 1996; Jensen et al. 2000; Rossi et al. 2000; Jabaudon et al. 2000; Sontheimer 2008). A third is via xCT as explained above. A fourth mechanism that has been vividly debated over the last decade is whether mature brain astrocytes in situ also have the ability to release glutamate by exocytosis (Bezzi et al. 2004). The differences in opinions can to some extent be explained by the use of different model systems. For instance, primary astrocytes in culture differ from mature astrocytes in the brain (Cahoy et al. 2008) so observations from cultures are not necessarily valid for the intact living brain. Nevertheless, it seems likely that also mature astrocytes in situ may release glutamate (Malarkey and Parpura 2008; Nedergaard and Verkhratsky 2012; Wang et al. 2013), but exocytosis of vesicles similar to those in nerve terminals is questionable (Hamilton and Attwell 2010). In fact, a recent paper refutes the notion that astrocytes express vesicular glutamate transporters (Li et al. 2013). Thus, this does not entirely rule out the concept of gliotransmitters because glutamate may be released via other mechanisms as explained above, but it does suggest critical evaluation of the literature.

## Regulation of the EAAT-type of transporters

Considering the importance of the glutamate transporters, pharmacological manipulation of transporter function may prove to be highly interesting from a therapeutic point of view (Sheldon and Robinson 2007). Although there are several examples where dysregulation of transporters contributes to the pathogenetic process, there are few examples of transporters being the primary cause (e.g. Danbolt 2001; Sattler and Rothstein 2006; Lauriat and Mcinnes 2007; Bröer and Palacin 2011). For instance, it is clear that complete absence of EAAT2 results in spontaneous epilepsy (Tanaka et al. 1997) and increased extracellular glutamate (Mitani and Tanaka 2003; Takasaki et al. 2008), but studies of humans with epilepsy have not uncovered any direct link to glutamate transporter expression (Tessler et al. 1999; Akbar et al. 1997; Bjørnsen et al. 2007). Nevertheless, studies from knockout mice and from humans with mutated transporters show links to disease (for a recent short update see Zhou and Danbolt 2013). Consequently, uncovering regulatory mechanisms is something that has been a hot topic and has interested a large number of researchers. A full account is beyond the scope of this review. Here we only mention a few points.

The first observation revealing regulation of glial glutamate transporter expression came from lesion experiments (Levy et al. 1995). Unilateral ablation of the neocortex in young adult rats resulted in ipsilateral down regulation of EAAT1 and EAAT2 in the striatum. The lesions did not penetrate the corpus callosum so striatum was not directly affected. However, the neocortical lesion eliminated the cell bodies that are responsible for the corticostriatal axons resulting in a loss of glutamatergic terminals in the striatum. Because astrocytes reduced their levels of EAAT1 and EAAT2 in response to the removal of these terminals, it was assumed that neuro-glia interactions were important in the regulation of transporter expression (Levy et al. 1995). This was followed up in cell cultures. Astrocytes cultured in the absence of neurons hardly expressed EAAT2 at all, while addition of neuron conditioned medium turned on EAAT2 expression (e.g., Gegelashvili et al. 1996, 1997, 2000, 2001; Plachez et al. 2000). This regulation turned out to be via several different pathways. Further, glutamate transporters are regulated by protein kinase C (Casado et al. 1993; reviewed by: Gonzalez and Robinson 2004; Vandenberg and Ryan 2013), by zinc (Vandenberg et al. 1998; Mitrovic et al. 2001; Vandenberg and Ryan 2013), and by arachidonic acid as mentioned above. In fact, there is regulation on more or less all levels from transcription to posttranslational modification and trafficking (for review see Seal and Amara 1999; Bergles et al. 1999; Hediger 1999; Kullmann 1999; Sims and Robinson 1999; Danbolt 2001; Robinson 2006; Sattler and Rothstein 2006). The most exciting discovery so far from a drug development point of view is the finding that beta-lactam antibiotics, e.g. Ceftriaxone, increase EAAT2 expression (Rothstein et al. 2005; Berry et al. 2013). Another team has also started high-throughput screening in order to identify translational activators of glial glutamate transporter EAAT2 (Colton et al. 2010) and have identified some pyridazine derivatives that may serve as lead compounds for drug development (Xing et al. 2011). Another interesting finding is a spider toxin that enhances EAAT2 transport activity (Fontana et al. 2007), but the compound responsible has not yet been identified.

## Approaches used to localize glutamate transporters

Early attempt to localize glutamate uptake sites were done using autoradiography in combination with tissue slices or synaptosome preparations (e.g. Minchin and Beart 1975; McLennan 1976; Beart 1976; Storm-Mathisen 1981; Storm-Mathisen and Wold 1981). To obtain higher resolution, thinner sections were needed. By using dry mount autoradiography (Young and Kuhar 1979; Danbolt et al. 1993) in combination with “sodium-dependent binding” of excitatory amino acids the uptake sites (for references see Danbolt 1994), higher resolution seemed to be within reach. However, heteroexchange complicated the interpretations as the amount of retained radioactively labeled ligand was dependent on both the number of transporter molecules and by the amount of endogenous dicarboxylic amino acid trapped within the membranes (Danbolt and Storm-Mathisen 1986a, b; Danbolt 1994).

From the early days of glutamate research, it was believed that glutamate is taken up by glutamatergic nerve terminals (Fonnum 1984), but the finding that glial glutamate transporters are down-regulated after glutamatergic denervation (Levy et al. 1995), weakened the evidence (for a discussion, see sect. 4.2 in Danbolt 2001). By incubating tissue slices in d-aspartate and fixing the slices, it was possible to detect fixed d-aspartate with antibodies. With this technique, uptake in both astrocytes and nerve terminals was demonstrated at the electron microscopic level (Gundersen et al. 1993). d-aspartate is often used instead of l-glutamate as a probe for glutamate uptake because it is slowly metabolized in brain tissue (Davies and Johnston 1976).

After the protein sequences of the transporters were known, synthetic peptides could be used to generate antibodies to the transporters themselves (Danbolt et al. 1998) rather than to the substrates. This led to an explosion in the use of antibodies to transporters, but, unfortunately, not all investigators validated their antibodies and procedures well enough (for detailed discussion see Holmseth et al. 2005, 2006, 2012a). The most difficult part is to obtain good negative controls. Antibodies may react with seemingly unrelated proteins (Holmseth et al. 2005; Zhou et al. 2014). In fact, antibody binding can always be achieved (see for instance Fig. 3 in: Holmseth et al. 2005). This is just a question of adjusting the assay conditions. Without a good negative control (e.g. tissue from knockout mice processed in parallel with tissue from wild-type mice), it is not possible to prove that the binding is to the antigen of interest. Therefore, antibody binding does not in itself prove that a given antigen is present. In this context it should be noted that the so called pre-adsorption test can easily give a false impression of specificity (Holmseth et al. 2012a). Whenever possible, it is a good idea to use additional methods, including in situ hybridization and Western blotting in combination with immunocytochemistry. TaqMan Real Time PCR is an excellent method for getting a first approximation of expression levels (e.g. Lehre et al. 2011; Zhou et al. 2012a). Another approach is to search available transcriptome and proteome datasets. For instance, proteome data from rat proximal tubules (http://dir.nhlbi.nih.gov/papers/lkem/pttr/) confirms the presence of EAAT3, but does not confirm expression of any of the other EAATs. Similarly, EAAT2 is in liver, but the other EAATs were not detected (http://141.61.102.16/), and neither the EAATs nor the VGLUTs were detected by proteome analysis of mouse pancreas (Zhou et al. 2014). Together, these data cast doubt over a large number of immunocytochemistry reports. The reason is obvious. Labeling with antibodies can always be obtained, and without good negative controls, it is not possible to tell if the labeling represents the antigen of interest or artifacts (see Holmseth et al. 2012a). Further, rapid post mortem proteolysis represents and additional challenge when studying human samples (Beckstrøm et al. 1999; Tessler et al. 1999; Li et al. 2012). Also note that water soluble proteins present in the samples may inhibit binding of transporters to the blotting membranes (Zhou et al. 2012b). Thus, strong upregulation of other proteins should be considered a potential source of error when estimating transporter levels by immunoblotting. Electron microscopy in combination with pre-embedding immunocytochemistry without detergents on unfrozen tissue is ideal for identification of labeled cell types, but is not ideal for subcellular distribution as the peroxidase reaction product diffuses some distance before precipitating. (Depending on the strength of the reaction, the reaction product may diffuse a couple of hundred nanometers.) In contrast, post-embedding immunogold is better for collecting semi-quantitative data and gives better intracellular resolution, but when cell membranes are labeled and cells are close to each other as they typically are in the brain, then immunogold cannot tell which membrane labeling belongs to (for description of these methods, see Danbolt et al. 1998; Amiry-Moghaddam and Ottersen 2013). Another problem with post-embedding immunogold is that there must be a sufficient number of target molecules in the plane of the section. This is case for EAAT1, EAAT2 and EAAT4. These proteins are present at very high concentrations (Dehnes et al. 1998; Lehre and Danbolt 1998) making them ideal targets for immunogold investigations. This explains, in part, why our early localization studies were so successful (Chaudhry et al. 1995; Dehnes et al. 1998). In contrast, EAAT3 is expressed at lower levels resulting in too few molecules per micrometer plasma membrane length to distinguish real labeling from background noise (Holmseth et al. 2012b). It should be recalled that the tissue sections used for electron microscopy are thin (40–60 nm) and thereby only slightly thicker than the outer diameter of synaptic vesicles (40 nm), and only two/three times thicker than the width of the synaptic cleft (20 nm). The antibodies do not penetrate well into the sections. To maximize labeling, the section may be mounted so that they can be labeled on both sides. Thus, the sensitivity of the post-embedding immunogold technique is limited by the number of proteins in the exact section plane. Another challenge follows from the vulnerability of the sections and thereby also the labeling. These sections are easily damaged during processing. Consequently, there is variability and this leads to another challenge: avoiding sampling error. This challenge comes in addition to those mentioned above (specificity, proteolysis, etc.).

It is also important to consider if any detected proteins are expressed at physiologically relevant levels. The number of molecules needed to accomplish a given task depends on what that task is. This consideration is particularly relevant for neurotransmitter transporters because the transport process is fairly slow. The cycling time of EAAT2 and EAAT3 are in the order of 30 glutamate molecules per second at Vmax (Otis and Jahr 1998; Otis and Kavanaugh 2000; Bergles et al. 2002; Grewer and Rauen 2005) and EAAT5 is even slower (Gameiro et al. 2011). The cycling time of the GABA transporters appear to be comparable to those of the EAATs (Mager et al. 1993; Sacher et al. 2002; Karakossian et al. 2005; Gonzales et al. 2007). This means that the number of transporters must be high. There is a rapid extracellular turnover of glutamate (Jabaudon et al. 1999), and despite this, the resting levels of extracellular glutamate in normal brains are low, possibly as low as 25 nm (Herman and Jahr 2007). Because the km-values are about 1,000 times higher (Danbolt 2001), maintenance of such low extracellular levels implies a vast excess of transporter proteins (Bergles and Jahr 1997; Dehnes et al. 1998; Lehre and Danbolt 1998; Otis and Kavanaugh 2000).

## Cellular and subcellular distribution of glutamate transporters in normal mature brain tissue

**Fig. 1:**
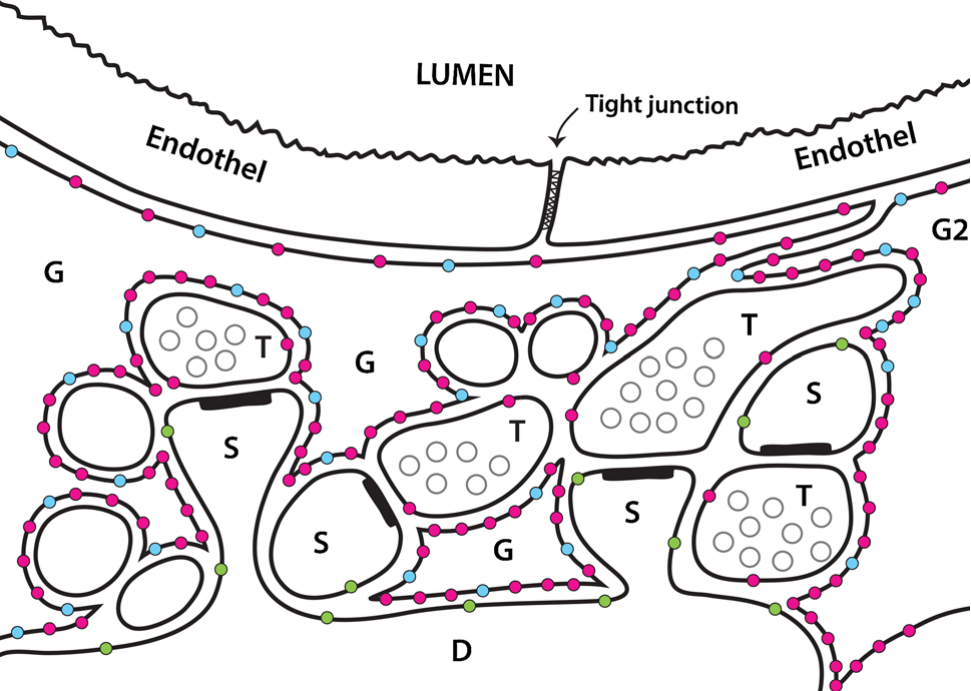
A schematic illustration of glutamate transporter distributions around synapses close to a blood vessel in the hippocampus. Four glutamatergic nerve terminals (*T*) are shown forming synapses onto dendritic spines (*S*). Astrocyte branches are indicated (*G*). Note that astrocytes have very high densities (Lehre et al. 1995; Ginsberg et al. 1995; Lehre and Danbolt 1998) of both EAAT2 (*red dots*) and EAAT1 (*blue dots*). The highest densities of EAAT1 and EAAT2 are in the astrocyte membranes facing neuropil, while the membranes facing the endothelium have low levels. Also note that glutamate transporters have not been detected in the endothelium. EAAT1 is selective for astrocytes (Lehre et al. 1995; Ginsberg et al. 1995), while EAAT2 is predominantly expressed in astrocytes (Danbolt et al. 1992), but there is also some (about 10 %) in hippocampal nerve terminals (Furness et al. 2008). EAAT3 (*green dots*) is selective for neurons, but is expressed at levels two orders of magnitude lower than EAAT2 and is targeted to dendrites and cell bodies (Holmseth et al. 2012b). Also note that the endfeet may actually overlap with no gaps in between them (Mathiisen et al. 2010) (Copyright: Neurotransporter AS; Reproduced with permission)

A large number of papers on transporter distributions have been published, and it is not easy to navigate in the literature as many of the statements are corrected in later publications. Figure 1 is a schematic illustration of the distributions of EAAT1, EAAT2 and EAAT3 in the forebrain.

EAAT1 (GLAST; slc1a3) is selectively expressed in astrocytes throughout the CNS (Lehre et al. 1995). This conclusion is supported both by in situ hybridization and immunocytochemistry (e.g. Ginsberg et al. 1995; Rothstein et al. 1995 Schmitt et al. 1997; Berger and Hediger 1998, 2000) and appears to be valid for all parts of the central nervous system including the regions where EAAT1 is the predominant transporter (Lehre et al. 1995; Lehre and Danbolt 1998; Takatsuru et al. 2007; Takayasu et al. 2009): the retina (Rauen et al. 1996; Lehre et al. 1997; Rauen et al. 1998; Rauen 2000; Rauen and Wiessner 2000), the inner ear (Furness and Lehre 1997; Takumi et al. 1997), and the circumventricular organs (Berger and Hediger 2000). Thus, there is no disagreement here. Other statements can be found in the literature, but these have been corrected by the authors themselves.

After having determined the cell types expressing EAAT1 (Lehre et al. 1995), immunogold was performed to obtain additional information (Chaudhry et al. 1995). This revealed that EAAT1 is preferentially targeted to the plasma membranes, and that plasma membranes facing neuropil have higher densities than those facing cell bodies, pia mater and endothelium (Fig. 1).

Mice lacking EAAT1 (Watase et al. 1998) develop normally, but show symptoms of insufficient glutamate uptake in regions where EAAT1 is the major glutamate transporter (Watase et al. 1998; Hakuba et al. 2000; Harada et al. 1998). The EAAT1 knockout mice also display poor nesting behavior; abnormal sociability, reduced alcohol intake and reward (Watase et al. 1998; Stoffel et al. 2004; Karlsson et al. 2009, 2012). Lack of GLAST does not lead to spontaneous seizures like those seen in connection with EAAT2-deficiency (Tanaka et al. 1997), but GLAST deficiency increases seizure duration and severity (Watanabe et al. 1999). EAAT1 mutations in humans are linked to episodic ataxia (Bröer and Palacin 2011; Jen et al. 2005; de Vries et al. 2009).

EAAT2 (GLT-1; slc1a2) was the first glutamate transporter to be localized immunocytochemically. In the mature and normal brain it is predominantly expressed in astrocytes (Danbolt et al. 1992; Levy et al. 1993; Rothstein et al. 1994; Lehre et al. 1995). There is no disagreement here either, and this conclusion is supported both by later immunocytochemistry (e.g. Schmitt et al. 1996; Kugler and Schmitt 2003; Berger et al. 2005; Holmseth et al. 2009) and in situ hybridization (Torp et al. 1994, 1997; Berger and Hediger 2000, 2001) as well as by data obtained with EAAT2 eGFP BAC reporter mice (de Vivo et al. 2010a).

EAAT2 is the only one of the EAAT-type of glutamate transporters that is required for survival under non-challenging conditions (Tanaka et al. 1997; Danbolt 2001). This is in agreement with biochemical data showing that the EAAT2 protein represents about 1 % of the total forebrain protein and that it is about four times more abundant than EAAT1 in the hippocampus and six times less abundant than EAAT1 in the cerebellum (Lehre and Danbolt 1998). Based on immunoadsorption of transport activity EAAT2 was shown to account for 95 % of the total glutamate uptake activity in young adult forebrain tissue (Danbolt et al. 1992; Haugeto et al. 1996). This conclusion was confirmed by deletion of the EAAT2 gene in mice (Tanaka et al. 1997; Voutsinos-Porche et al. 2003; Matsugami et al. 2006; Kiryk et al. 2008; Holmseth et al. 2012b) as well as by electrophysiological recordings of glutamate transporter currents (Otis and Kavanaugh 2000).

The discussion about EAAT2 distribution concerns expression in neurons. Having said that, there is consensus that EAAT2 is expressed in cultured neurons from hippocampus and neocortex; in particular if these are cultured in the absence of astrocytes (Mennerick et al. 1998; Wang et al. 1998; Plachez et al. 2000) in agreement with observations that EAAT2 is transiently localized on growing axons of the mouse spinal cord before establishing astrocytic expression (Yamada et al. 1998). There is also consensus that EAAT2 is present in neurons in the normal and mature mammalian retina (Rauen et al. 1996, 1999; Rauen and Kanner 1994; Euler and Wassle 1995; Rauen 2000).

The controversy is related to expression of EAAT2 in neurons in the normal and mature brain (cerebrum and cerebellum). All studies, however, agree that there is EAAT2 mRNA in CA3 hippocampal neurons (Torp et al. 1994, 1997; Berger and Hediger 2000, 2001; de Vivo et al. 2010a) and that their axon-terminals express the protein, at least in the CA1 (Chen et al. 2004; Furness et al. 2008; Melone et al. 2009, 2011). Further, all of the glutamate uptake activity in glutamatergic terminals in CA1 is due to EAAT2 (Furness et al. 2008).

The remaining controversy concerns (a) the expression of EAAT2 in axon-terminals in other parts of the brain, and (b) the physiological importance of the uptake into terminals. Why was about half of all d-aspartate taken up by hippocampus slices found in axon-terminals when terminals only contain around 10 % of the EAAT2 protein (Furness et al. 2008)? This disproportionally large uptake cannot simply be disregarded as an in vitro artifact due to a higher rate of heteroexchange than net uptake (Zhou et al. 2013), but it might still be an artifact because the possibility has not been ruled out that astrocytes release glutamate via anion channels or similar. Preliminary data from selective deletion of EAAT2 in axon-terminals indicate disturbances in synaptic transmission (Sun et al. 2012), and thereby may suggest that EAAT2 in terminals is functionally relevant. However, further studies are required before definite conclusions can be made.

In contrast to EAAT1, there is very little EAAT2 in mice and rats at birth and in the first postnatal week (Ullensvang et al. 1997; Furuta et al. 1997). This explains why EAAT2-knockout mice are inconspicuous at birth. But at 3 weeks, when the EAAT2-levels in wild-type mice have increased to 50 % of adult levels, the EAAT2-deficient mice can readily be identified because they are hyperactive, epileptic and smaller than their wild-type littermates. They have increased extracellular glutamate levels (Mitani and Tanaka 2003; Takasaki et al. 2008), and about half of them die from spontaneous seizures before they reach 4 weeks of age (Tanaka et al. 1997). The heterozygote EAAT2 knockout mice (±) have only half the EAAT2-concentrations as wild-type mice, but do not show any apparent morphological brain abnormalities (Kiryk et al. 2008), but are more vulnerable to traumatic spinal cord injury (Lepore et al. 2011).

EAAT3 (EAAC1; slc1a1) has been particular hard to localize. Nevertheless, the first studies were basically correct (Kanai and Hediger 1992; Rothstein et al. 1994). EAAT3 is a neuronal transporter, and is not expressed in glial cells (Holmseth et al. 2012b; Shashidharan et al. 1997). It appears to be expressed in the majority if not all neurons throughout the CNS, but has a unique sorting motif (Cheng et al. 2002) selectively targeting it to somata and dendrites avoiding axon terminals (Holmseth et al. 2012b; Shashidharan et al. 1997).

The highest levels of EAAT3 in the brain are found in the hippocampus and neocortex, but the total tissue content in young adult rat brains is about 100 times lower than that of EAAT2 (Holmseth et al. 2012b). It is also expressed in the kidney and in the ileum. In agreement, mice lacking EAAT3 (Peghini et al. 1997) develop dicarboxylic aminoaciduria, but do not show signs of neurodegeneration at young age and do not have epilepsy (Peghini et al. 1997; Aoyama et al. 2006; Berman et al. 2011). Humans lacking EAAT3 develop dicarboxylic aminoaciduria (Bailey et al. 2011) and EAAT3 polymorphisms are associated with obsessive–compulsive disorders (Brandl et al. 2012; Walitza et al. 2010).

EAAT4 (slc1a6) is predominantly found in the cerebellar Purkinje cells (Fairman et al. 1995; Dehnes et al. 1998) where it is targeted to the dendrites, the spines in particular (Dehnes et al. 1998), but there is also some EAAT4 in a subset of forebrain neurons (Dehnes et al. 1998; Massie et al. 2008; de Vivo et al. 2010b) and in vestibular hair cells and calyx endings (Dalet et al. 2012). EAAT4 knockout mice are viable and appear normal (Huang et al. 2004) albeit with some alteration of receptor activation (Nikkuni et al. 2007).

EAAT5 (slc1a7) is preferentially expressed in the retina, while the levels in the brain are low (Arriza et al. 1997; Eliasof et al. 1998). EAAT5 is also expressed in vestibular hair cells and calyx endings (Dalet et al. 2012). There is more than one isoform in the retina due to variable splicing (Eliasof et al. 1998). As explained above, EAAT4 and EAAT5 are not very efficient as transporters, but are efficient chloride channels suggesting that they may be more important as inhibitory glutamate receptors than as transporters. Some investigators have tried to determine the exact cellular and subcellular localization of EAAT5, but the validity of these studies is hard to judge at present because nobody has as yet made an EAAT5 knockout mouse that could serve as negative control for validation of the immunolabeling. We have previously shown how important this control is and also how inadequate the so called pre-adsorption test is (Holmseth et al. 2012a). So, validated information on EAAT5 distribution remains to be provided.

## Comments on the glutamine-glutamate cycle

Glutamate taken up by astroglial cells can be metabolized via the tricarboxylic acid cycle and be used in protein synthesis or converted to glutamine. Glutamine can be released to the extracellular fluid by a sodium neutral amino transporter in the astrocytic membrane by SNAT3 (Boulland et al. 2002, 2003; Mackenzie and Erickson 2004; Nissen-Meyer et al. 2011) and SNAT5 (SN2; slc38a5) (Hamdani et al. 2012) because it is inactive in the sense that it cannot activate glutamate receptors (for review: Erecinska and Silver 1990; Danbolt 2001; Hertz 2013). The conversion of glutamate to glutamine is catalyzed by the enzyme glutamine synthetase (GLUL) in an ATP-dependent manner (Erecinska and Silver 1990; Marcaggi and Coles 2001). Glutamine synthetase plays important roles in the brain and in other organs from implantation to high age. This is evident from studies of glutamine deficiency in man and mice (He et al. 2007, 2010a, b; Haberle et al. 2011, 2012). Further, reduced glutamine synthetase levels are associated with some forms of epilepsy (Eid et al. 2004).

The prevailing view has been that glutamine from astrocytes is the predominant source of glutamate in glutamatergic terminals (Sibson et al. 2001; Hertz 2013), but this hypothesis implies that the supply of glutamine to terminals keeps up with glutamate release. And although there are many observations in cultured cells suggesting the existence of glutamine transporters in glutamatergic terminals, it is important to keep in mind that cultured astrocytes are different from mature astrocytes (e.g. Plachez et al. 2000; Cahoy et al. 2008). Further, it is important to note that glutamine transporters have so far not been positively identified in terminals in brain tissue (Mackenzie and Erickson 2004; Chaudhry et al. 2002; Conti and Melone 2006). The only positive identifications of SNAT2 (SAT2; slc38a2) and SNAT1 (SAT1; GlnT; slc38a1) are in dendrites and cell bodies of neurons (e.g. Jenstad et al. 2009; Solbu et al. 2010; Conti and Melone 2006). One possibility is that they have evaded detection in glutamatergic terminals due to methodological challenges. Another possibility is that they have not been detected simply because they are not there. This would be in line with studies suggesting that SNAT1 and SNAT2 play no role in delivering glutamine for glutamatergic transmission (Grewal et al. 2009). There could be other glutamine transporters, however. For instance, ASCT2 (slc1a5) has ability to transport glutamine (Bröer et al. 1999), but is expressed at low levels in the mature brain (Utsunomiya-Tate et al. 1996; Bröer and Brookes 2001). There are also other potential candidates within the slc38-family. On the other hand, lack of significant glutamine uptake activities in terminals would be is in line with some old reports (e.g. Hertz et al. 1980; Yu and Hertz 1982; McMahon and Nicholls 1990). Another possibility is whether glutamate may be formed in a glutamine-independent manner (Hassel and Bråthe 2000; McKenna et al. 2000), but this is also debated. A third source is direct uptake by glutamate transporters in terminals themselves (Gundersen et al. 1993). As explained above, there is EAAT2 in terminals and this uptake is highly active (Furness et al. 2008). Another complicating factor is that nerve terminals in different brain regions may differ. While terminals in several forebrain regions (e.g. neocortex, hippocampus and striatum) have been shown to posses glutamate uptake activity (e.g. Gundersen et al. 1993), this is more uncertain in the cerebellar cortex (e.g. Wilkin et al. 1982). In conclusion, the glutamine-glutamate cycle has been studied and debated for about 50 years and we still do not have the final answer!

## Glutamate transporters at the blood brain barrier

The nervous system isolates itself from blood by means of barriers (e.g. Abbott 2005; Alvarez et al. 2013). This is important for a number of reasons. One of them is the fact that serum glutamate is typically in the range 50–200 μm (Zlotnik et al. 2011a, b, c) which is orders of magnitude higher than the concentrations that are toxic to neurons (Danbolt 2001).

The blood–brain barrier is between blood and the interstitial fluid of the brain. It is in mammals formed by the endothelial cells after influence from brain cells. Another barrier is in the choroid plexus epithelium which secretes cerebrospinal fluid (CSF). These barriers are important both from a physiological point of view because they are essential for brain homeostasis, and from a pharmacological point of view because they prevent drugs from entering brain tissue (Deboer and Gaillard 2007; Teichberg 2007). The literature is extensive and full of conflicting reports. A full account is beyond the scope of this review. Here we only want to point out (Fig. 1) that brain barrier endothelial cells do not express significant levels of EAAT1-3 (Lehre et al. 1995; Berger and Hediger 2000; Holmseth et al. 2009, 2012b). There are, however, huge amounts of glutamate transporters in the astrocytic endfeet surrounding the blood vessels (Fig. 1). When isolating brain microvessels, the preparations are likely to be contaminated by endfeet and this may explain some of the data. Thus, it seems that no significant transport of glutamate can occur through a normal and intact blood–brain barrier. In agreement, injection of radiolabeled glutamate and aspartate does not result in accumulation of radioactivity in the brain (Klin et al. 2010). On the other hand, there is an efflux mechanism for glutamate as blood-mediated scavenging is reported to reduce glutamate in the cerebrospinal fluid (Gottlieb et al. 2003). There is some evidence that this may offer some protection (Zlotnik et al. 2008; Teichberg et al. 2009; Zlotnik et al. 2010; Nagy et al. 2010). The mechanism, however, of release from the brain remains to be identified. This illustrates that brain water homeostasis and transport mechanisms between the blood and the extracellular fluid in brain are incompletely understood. Recent work from Nedergaard and co-workers may represent a leap in our understanding. They introduce the term “glymphatics” (Iliff et al. 2012; Nedergaard 2013) to describe flow of fluid from the arachnoid space along blood vessels into brain tissue. This may reconcile a number of apparently conflicting reports. Perhaps this also will explain why the betaine-GABA transporter (BGT1; slc6a12; Zhou et al. 2012b) and the taurine transporting GABA transporter 2 (GAT2; slc6a13; Zhou et al. 2012a) are expressed in the leptomeninges.

## Concluding remarks

As outlined above, substantial progress has been made over the last decades. But there are major gaps in our understanding of key processes. One example is transport of metabolites across the blood brain barrier. Another unknown is the uptake in glutamatergic nerve endings and the relevance of the glutamate-glutamine cycle for transmitter glutamate. A third topic is why the body needs several different glutamate transporters, and how they can be pharmacologically modulated.

## Abbreviations

AMPA: α-Amino-3-hydroxy-5-methyl-4-isoxazole propionic acid
l-AP4: l-2-Amino-4-phosphonobutanoate
ATP: Adenosine triphosphate
CNS: Central nervous system
EAAC1: Glutamate transporter number 3 (EAAT3; slc1a1; Kanai and Hediger 1992)
EAAT: Excitatory amino acid transporter (synonymous to glutamate transporter)
GABA: γ-Aminobutyric acid
GLAST: Glutamate transporter number 1 (EAAT1; slc1a3; Storck et al. 1992; Tanaka 1993a)
GLT-1: Glutamate transporter number 2 (EAAT2; slc1a2; Pines et al. 1992)
GLUL: Glutamine synthetase
NMDA: *N*-Methyl-d-aspartate
TBOA: dl-*threo*-β-benzyloxyaspartate
